# Cytotoxic Activity of *Opopanax hispidus* (Friv.) Griseb.: Characterization of a New Chalcone‐type Compound

**DOI:** 10.1002/cbdv.202501077

**Published:** 2025-07-22

**Authors:** Kevser Taban, İpek Süntar, Perihan Gürbüz, Esra Emerce, Osman Tugay, Şengül Dilem Doğan, Beyza Hamur, Ahmet Ceyhan Gören

**Affiliations:** ^1^ Department of Pharmacognosy, Faculty of Pharmacy Sivas Cumhuriyet University Sivas Türkiye; ^2^ Department of Pharmacognosy, Faculty of Pharmacy Gazi University Ankara Türkiye; ^3^ Department of Pharmacognosy, Faculty of Pharmacy Erciyes University Kayseri Türkiye; ^4^ Department of Pharmaceutical Toxicology, Faculty of Pharmacy Gazi University Ankara Türkiye; ^5^ Department of Pharmaceutical Botany, Faculty of Pharmacy Selcuk University Konya Türkiye; ^6^ Department of Basic Sciences, Faculty of Pharmacy Erciyes University, Kayseri Türkiye; ^7^ Department of Chemistry, Faculty of Basic Sciences Gebze Technical University Gebze, Kocaeli Türkiye; ^8^ Troyasil HPLC Column Technologies Doruk Analitik, Mehmet Akif Mah. Yumurcak Sok. No:43 Ümraniye İstanbul Türkiye

**Keywords:** Apiaceae, chalcone, cytotoxic activity, lung cancer, *Opopanax hispidus*

## Abstract

The present study aimed to investigate cytotoxic compounds of *Opopanax hispidus* (Friv.) Griseb. through in vitro, in silico, and phytochemical analyses. The cytotoxic activity of *O. hispidus* was evaluated against four different cancer cell lines – lung (A549), breast (MCF‐7), liver (HepG2), and cervix (HeLa) – as well as one healthy cell line (Beas‐2B), using the 3‐(4,5‐dimethylthiazol‐2‐yl)‐2,5‐diphenyltetrazolium bromide colorimetric assay. *n‐*Hexane and dichloromethane sub‐extracts with the IC_50_ values of 9.77 ± 0.57 and 7.10 ± 0.78 mg/mL on A549, respectively, were fractionated through chromatographic techniques, and the structures of the isolated compounds were elucidated using various spectroscopic methods. A novel compound, 2',6'‐dihydroxy‐4'‐methoxy‐3''‐(ɤ,ɤ‐dimethylallyl)‐4''‐hydroxy *β*‐hydroxydihydrochalcone (**3**), along with three known coumarins – umbelliferone 6‐carboxylic acid (**1**), umbelliferone (**2**), and nodakenetin (**4**) – were isolated from the active fractions. Compound **3**, which possesses a chalcone structure, exhibited notable cytotoxicity against lung cancer cells with an IC_50_ value of 13.72 ± 0.80 µM.

## Graphical abstract

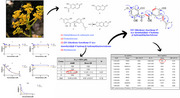

## 1 Introduction

Cancer, characterized by uncontrolled cell growth and abnormal signaling processes, is one of the most life‐threatening diseases. Its global incidence is on the rise, emphasizing the urgent need for the discovery of novel compounds and therapeutic strategies to combat cancer. One of the primary challenges in cancer treatment is the toxicity of anticancer drugs to healthy cells, which results in numerous adverse effects. The structural diversity and bioactivity potential of natural compounds present promising opportunities for developing new anticancer agents targeting key cancer types [1]. Cytotoxicity‐guided studies serve as an essential initial step in identifying compounds that can target tumors through various mechanisms, such as alkylating agents, mitotic inhibitors, and topoisomerase inhibitors.

The Apiaceae family, which includes a wide array of economically and medicinally valuable species, comprises approximately 450 genera and over 3500 species worldwide [2]. Numerous species within this family, particularly those from the genera *Ferula* L., *Smyrnium* L., *Prangos* Lindl., *Ferulago* W. Koch, *Pimpinella* L., *Heracleum* L., *Angelica* L., *Eryngium* L., and *Peucedanum* L., have been studied for their anticancer properties. The active compounds responsible for these effects are predominantly flavonoids, terpenoids, and coumarins [3, 4, 5, 6, 7]. Based on the literature, it was hypothesized that plants from the Apiaceae family could serve as potential sources for anticancer research. Therefore, this study focused on *Opopanax hispidus* (Friv.) Griseb., from the Apiaceae family, a species for which detailed phytochemical and cytotoxicity studies have yet to be conducted.

The term Opopanax is a source of confusion, as it refers to distinct substances that share the same name. Similar to olibanum and myrrh, Opopanax designates a resin obtained from specific Commiphora shrub species, which are unrelated to the genus *Opopanax* W.D.J. Koch and are distributed across regions from the Middle East to Africa [8]. In contrast, the genus *Opopanax* W.D.J. Koch is primarily found in temperate climates. The precise origin of the so‐called “true *Opopanax*” remains uncertain due to the ambiguous descriptions provided by ancient authors [9].

The genus *Opopanax* is represented by four species in Türkiye, namely *Opopanax hispidus* (Friv.) Griseb., *Opopanax chironium* (L.) W. Koch, *Opopanax persicus* Boiss. and *Opopanax siifolius* (Boiss. & Heldr.) Menemen [10]. These species have traditionally been used for their various medicinal properties, including cancer. For instance, ointment with a mixture of essential oil, gum, and resin obtained from *O. chironium* has been used to treat different cancers [11]. Moreover, heraclenin and imperatorin from *O. chironium* were determined to induce apoptosis in leukemia cells and show selective cytotoxicity toward cancer cells [12].

*O. hispidus*, known locally as “kekire, kaymecik, gaymecik” [13, 14, 15], has a historical use as an antidote in Dynameron [16]. The stems, leaves, and flowers of *O. hispidus* were used for antiseptic purposes in Iran. In Türkiye, fresh stems are used to treat female infertility; leaves are used against hemorrhoids [15, 17, 18]. Moreover, fresh stems and basal leaves are consumed as food in some parts of Türkiye [17].

Several bioactive compounds from *O. hispidus* have been described in the literature, demonstrating anti‐inflammatory, antibacterial, antioxidant, and enzyme‐inhibitory properties, particularly in relation to infertility [8, 10, 19, 20, 21]. The essential oil of *O. hispidus* contains labdane‐type diterpenes and sesquiterpenes, with torulosol, geranyl geraniol acetate, and germacrene D as major components [22]. Additionally, the flowers and leaves of *O. hispidus* are rich in phenolics and flavonoids [8]. However, isolation studies on this plant remain limited. Notably, seven coumarins—3'‐isobutyryl‐3'‐hydroxymarmesin, oreoselon, peucedanin, officinalin, smirniorin, 4'‐acetyl‐3'‐isobutyryl‐3'‐hydroxymarmesin, and 3'‐hydroxypranthimgin—were isolated from the chloroform extract of the aerial parts of *O. hispidus* [23].

In the present study, an in vitro activity‐guided fractionation assay was conducted to isolate cytotoxic secondary metabolites from the *n*‐hexane and dichloromethane sub‐extracts of the whole plant of O*. hispidus*. The cytotoxicity of these compounds was evaluated against lung (A549), breast (MCF7), liver (HepG2), and cervix (HeLa) cancer cells. Additionally, in silico predictions were performed to assess the biological activities of the isolated compounds and to support cytotoxicity results.

## 2 Results and Discussion

### 2.1 Structure Elucidation and Identification

**FIGURE 1:**
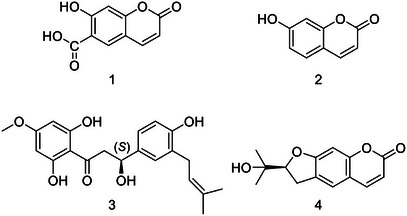
Structures of the compounds (**1**–**4**) isolated from the *O. hispidus*.

The crude methanol extract of *O. hispidus* was suspended with distilled water and partitioned with organic solvents of increasing polarity: *n*‐hexane, dichloromethane, ethyl acetate, and *n*‐butanol (see section 2.3). The OHNH and OHDCM subextracts were fractionated by repeated column chromatography (Sephadex LH‐20, silica gel, MPLC, and preparative TLC) to obtain a new *β*‐hydroxydihydrochalcone (**3**), and three known coumarins: umbelliferone 6‐carboxylic acid (**1**), umbelliferone (**2**), and nodakenetin (**4**) (Figure 1).

Although coumarin derivatives, umbelliferone, umbelliferone 6‐carboxylic acid, and nodakenetin, were isolated from different genera belonging to the Apiaceae family in the literature, their isolation from the *Opopanax* genus was reported for the first time in the present study.

**FIGURE 2:**
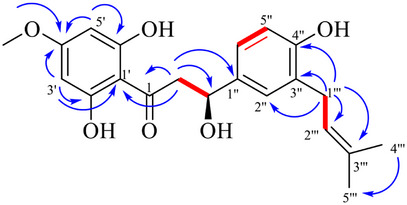
Key ^1^H‐^1^H COSY (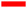) ve HMBC (H
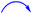
C) correlations for compound 3.

Compound **3** was isolated as a yellowish‐white solid. The molecular formula C_21_H_24_O_6_ was established based on the electrospray ionization (negative ion mode) molecular ion obtained at *m/z* 371.1457 [M─H]^‐^ (calcd. for C_21_H_23_O_6_, 371.1489). The ^1^H nuclear magnetic resonance (NMR) showed two doublet of doublets at *δ*_H_ 3.12 (dd, *J* = 17.1, 12.6 Hz, H‐2a) and *δ*_H_ 2.73 (dd, *J* = 17.1, 3.1 Hz, H‐2b) indicates the two diasterotopic protons of methylene group and ^13^C NMR *δ*_C_ 198.3 (C═O), 44.0 (C‐2), and 80.8 (C‐3) data suggested the compound to have a β‐hydroxydihydrochalcone skeleton. The ^1^H NMR and ^1^H‐^1^H correlated spectroscopy (COSY) spectra of **1** revealed two aromatic rings. The first one showed 2,4,6‐trisubstitution ring systems and confirmed from the signals *δ*_H_ 6.05 (d, *J* = 2.3 Hz, H‐5ʹ) and *δ*_H_ 6.03 (d, *J* = 2.3 Hz, H‐3ʹ). The protons of the second aromatic ring revealed the ABX system at *δ*_H_ 7.17 (d, *J* = 2.2 Hz, H‐2ʹʹ), 7.13 (dd, *J* = 8.2, 2.2 Hz, H‐6ʹʹ), and 6.79 (d, *J* = 8.2 Hz, H‐5ʹʹ). Besides, the presence of those at *δ*_H_ 1.72 and 1.74 and at *δ*_C_ 133.2 and 123.7 indicated the ɤ,ɤ‐dimethylallyl group. The ^13^C NMR spectrum of the compound exhibited twenty‐one carbon resonances including a carbonyl carbon (*δ*_C_ 198.3), two methylene carbons (*δ*_C_ 44.0 and 29.3), a carbon bearing a hydroxyl group (*δ*_C_ 80.8), carbons belong to the ɤ,ɤ‐dimethylallyl group (*δ*_C_ 133.2, 123.7, 25.9, 17.9), one methoxyl group (*δ*_C_ 56.2) and aromatic carbons *δ*_C_ 169.5, 165.2, 164.7, 156.7, 130.8, 129.6, 129.0, 126.2, 115.7, 104.1, 95.7, and 94.9 (Table 1). The connectivity of the pronated carbons between C‐1ʹʹʹ and C‐2 ʹʹʹ and C‐2 and C‐3 was detected by the COSY spectrum of **3** (Figure 2).

**TABLE 1:** ^1^H and ^13^C nuclear magnetic resonance (NMR) data of compound **3**.

Position	C/H	*δ*_C_ ppm	*δ*_H_ ppm, *J* (Hz)
**1**	C	198.3	—
**2**	CH_2_	44.0	3.12 (H‐2a, dd, *J* = 17.1; 12.6 Hz)
			2.73 (H‐2b, dd, *J* = 17.1; 3.1 Hz)
**3**	CH	80.8	5.30‐5.37^*^ (1H, m)
**1ʹ**	C	104.1	—
**2ʹ**	C	165.2	—
**3ʹ**	CH	95.7	6.03 (1H, d, *J* = 2.4 Hz)
**4ʹ**	C	169.5	—
**5ʹ**	CH	94.9	6.05 (1H, d, *J* = 2.3 Hz)
**6ʹ**	C	164.7	—
**1ʹʹ**	C	130.8	—
**2ʹʹ**	CH	129.0	7.17 (1H, d, *J* = 2.2 Hz)
**3ʹʹ**	C	129.6	—
**4ʹʹ**	C	156.7	—
**5ʹʹ**	CH	115.7	6.79 (1H, d, *J* = 8.2 Hz)
**6ʹʹ**	CH	126.2	7.13 (1H, dd, *J* = 8.2 and 2.3‐2.2 Hz)
**1ʹʹʹ**	CH_2_	29.3	3.30^z^
**2ʹʹʹ**	CH	123.7	5.30‐5.37^*^(1H, m)
**3ʹʹʹ**	C	133.2	—
**4ʹʹʹ**	CH_3_	25.9	1.72 (3H, s)
**5ʹʹʹ**	CH_3_	17.9	1.74 (3H, s)
**4′‐OCH_3_**	CH_3_	56.2	3.81 (3H, s)

* Overlapped protons, ^z^ Overlapped with solvent signal.

The linkage position of the methoxy and dimethylallyl groups was established by observation of HMBC correlations. The HMBC spectrum showed that the methylene signal at *δ*_H_ 3.30 (H‐1′′′) was correlated with C‐3′′ (*δ*_C_ 129.6), C‐4′′ (*δ*_C_ 156.7), C‐2′′′ (*δ*_C_ 123.7), and C‐3′′′ (*δ*_C_ 133.2), indicating that the dimethylallyl group was attached to C‐3′′. Moreover, the methoxy signal at *δ*_H_ 3.81 (4′‐OCH_3_) was correlated with C‐4′ (*δ*_C_ 169.5), suggesting that the methoxy group was attached to C‐4′. From the above discussion, compound **3** was found to be a new *β*‐hydroxydihydrochalcone and given a trivial name as opopanaxchalcone.

**FIGURE 3:**
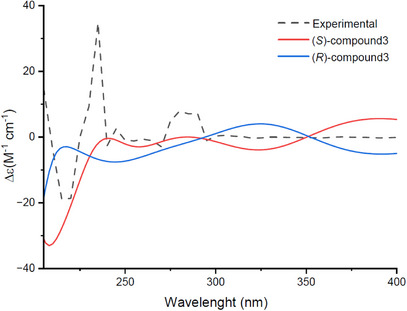
Experimental and computational electronic circular dichroism (ECD) spectra of compound **3**.

Based on the conformational analysis, the electronic circular dichroism (ECD) spectra of (*S*)‐compound **3** and (*R*)‐compound **3** were calculated in chloroform at the B3LYP/6‐311G(d,p) level using time‐dependent density functional theory (TDDFT). As shown in Figure 3, the experimental spectrum shows a strong negative Cotton effect around 220 nm and a positive one near 250 nm. These features are well reproduced by the calculated spectrum of the (*S*)‐enantiomer. In contrast, the (*R*)‐enantiomer shows opposite signs at these wavelengths. A weak shoulder around 285 nm is also better matched by the (*S*)‐compound **3**. Overall, the comparison confirms that compound **3** has the (*S*)‐absolute configuration.

*β*‐Hydroxydihydrochalcones are rare natural compounds. Ziganin, a glucosyldihydrochalcone from *Pimpinella rhodantha* Boiss. (Apiaceae); 3‐(*S*)‐hydroxy‐3‐phenyl‐1‐(2′,4′,6′‐trihydroxyphenyl)propan‐1‐one from *Myristica beddomei* subsp. *spherocarpa* W.J. de Wilde (Myristicaceae); balanochalcone from *Balanophora laxiflora* Hemsl. (Balanophoraceae); (*S*)‐elatadihydrochalcone from *Tephrosia elata* Deflers (Fabaceae); (*S*)‐elatadihydrochalcone‐2′‐methyl ether from *Tephrosia uniflora* Persia. (Fabaceae) were reported as new *β*‐hydroxydihydrochalcone derivatives [24, 25, 26, 27, 28]. In this study, opopanaxchalcone isolated from the dichloromethane sub‐extract of *O. hispidus* was reported as a novel *β*‐hydroxydihydrochalcone derivative.

### 2.2 Cytotoxic Activity

The cytotoxic activity of crude methanol extract was investigated on MCF‐7, HeLa, HepG2, A549 cancer cells, and Beas‐2B cells using the 3‐(4,5‐dimethylthiazol‐2‐yl)‐2,5‐diphenyltetrazolium bromide (MTT) assay. The extract showed the highest activities on MCF‐7 and A549 with IC_50_ values of 21.44 µg/mL and 40.78 µg/mL, respectively. Thus, the sub‐extracts obtained from the methanol extract were studied on lung and breast cancer cells (Figures S1 and S2). Among sub‐extracts, OHNH and OHDCM showed significant effects in a dose‐dependent manner with the IC_50_ values of 9.77 ± 0.57 and 7.10 ± 0.78 on A549, respectively. These two sub‐extracts had IC_50_ values of less than 20 µg/mL in both breast and lung cancer cells (Table 2). Ethyl acetate, *n*‐butanol, and water sub‐extracts showed no activity in the tested concentrations.

**TABLE 2:** Cytotoxic activity results of *O. hispidus* methanol extract and sub‐extracts against the tested cell lines.

Extract	IC_50_ ± SEM (µg/mL)				
	MCF‐7	A549	HeLa	HepG2	Beas‐2B
OH MeOH	21.44 ± 2.01	40.78 ± 2.54	49.53 ± 2.77	77.68 ± 5.84	36.82 ± 2.01
OHNH	10.90 ± 0.41	9.77 ± 0.57	—	—	6.42 ± 0.32
OHDCM	8.47 ± 0.95	7.10 ± 0.78	—	—	2.16 ± 0.19
OHEtOAc	>100	>100	—	—	>100
OHBuOH	>100	>100	—	—	>100
OHH_2_O	>100	>100	—		>100
Dox^*^	1.89 ± 0.27	0.82 ± 0.13	0.55 ± 0.06	3.02 ± 0.37	0.34 ± 0.02

* The results of doxorubicin were given as IC_50_ ± SEM (µM). *Dox: doxorubicin; SEM: standard error of the mean; OHNH: n‐hexane; OHDCM: dichloromethane; OHEtOAc: ethyl acetate; OHBuOH: n‐butanol; OHH_2_O: water*.

A dramatic increase in activity was noted in the OHNH and OHDCM sub‐extracts compared to OH MeOH. In breast cancer cells, the IC_50_ for OH MeOH was 21.44 µg/mL, and it was calculated as 10.90 and 8.47 µg/mL in OHNH and OHDCM, respectively. On the other hand, in the lung cancer cells, IC_50_ values were recorded as 40.78 µg/mL for OH MeOH, while for OHNH and OHDCM, it was 9.77 and 7.10 µg/mL, respectively. The cytotoxic activity potential of sub‐extracts against lung cancer was remarkable.

According to the World Health Organisation (WHO) report, lung cancer ranks first in cancer‐related deaths and second among the most diagnosed cancers [29]. In this context, the need to discover new strategies and new compounds in the treatment remains essential. Lung cancer cells were the most susceptible to the cytotoxicity of the *O. hispidus* sub‐extracts among the cancer cell lines employed in this study. Therefore, to identify potent cytotoxic compounds against lung cancer, further research was conducted on OHNH and OHDCM.

In activity‐guided fractionation studies, OHNH and OHDCM were fractionated using the Sephadex LH‐20 CC, and OHNH Fr1‐4 and OHDCM Fr1‐2 subfractions were obtained. Each fraction was studied for cytotoxicity against A549 and Beas‐2B cells (Figures S3 and S4). Except for OHNH Fr4, all fractions of OHNH exerted potent activity (IC_50_ = 6.89‐12.05 µg/mL), and OHNH Fr2 was the most active one (Table 3). The fractions of the dichloromethane subextract, OHDCM Fr1 and OHDCM Fr2, showed potent dose‐dependent cytotoxicity against A549 (IC_50_ = 4.92 and 3.20 µg/mL, respectively).

**TABLE 3:** Cytotoxic activity results of the *O. hispidus* subfraction of OHNH and OHDCM against A549 and Beas‐2B cell lines.

Fraction	IC_50_ ± SEM (µg/mL)		SI
	A549	Beas‐2B	
OHNH Fr1	8.20 ± 1.28	3.37 ± 0.20	0.41
OHNH Fr2	6.89 ± 0.63	4.23 ± 0.33	0.61
OHNH Fr3	12.05 ± 1.36	10.50 ± 1.24	0.87
OHNH Fr4	>100	28.03 ± 11.91	—
OHDCM Fr1	4.92 ± 0.52	4.49 ± 0.39	0.91
OHDCM Fr2	3.20 ± 0.41	2.28 ± 0.28	0.71
Dox^*^	0.59 ± 0.05	0.07 ± 0.017	0.12

* The results of doxorubicin were given as IC_50_ ± SEM (µM). *Dox: doxorubicin; SEM: Standard error of the mean; SI: selectivity index; OHNH: n‐hexane; OHDCM: dichloromethane*.

Following sequential chromatographic techniques on active OHNH Fr2 and OHDCM Fr2 sub‐extracts, four compounds were isolated, and their cytotoxic effects were tested on A549 and Beas 2B (Figure S5). Compound **3**, whose structure was elucidated for the first time in this study, exerted potent cytotoxicity against lung cancer cells (IC_50_ = 13.72 µM). The selectivity index of the compound **3** was calculated as 0.87. However, it was found to be 0.12 for the positive control, doxorubicin. The primary goal of finding an ideal anticancer compound is to develop a structure that targets tumors specifically while causing minimal or no harm to healthy cells. Although studies have been conducted with this approach, the drugs currently used in the clinics still face challenges. In the present study, for compound **3**, whose selectivity was less than 1, in addition to advanced anticancer research, different approaches may need to be pursued to reduce its toxicity. Today, the efforts are continued that selectivity can be improved by changing the formulation forms of compounds, using smart drug technologies, or varying the physicochemical properties of the compound [30, 31, 32]. Compounds with coumarin skeleton (**1**, **2**, and **4**) did not show an antiproliferative effect at the determined concentrations (Table 4).

**TABLE 4:** Cytotoxic activity results of the isolated compounds of *O. hispidus* against A549 and Beas‐2B cell lines.

Compound	IC_50_ ± SEM (µM)		SI
	A549	Beas‐2B	
**1**	>50	>50	—
**2**	>50	>50	—
**3**	13.72 ± 0.80	11.97 ± 1.5	0.87
**4**	>50	>50	—
**Dox**	0.59 ± 0.05	0.07 ± 0.017	0.12

*Dox: doxorubicin; SEM: standard error of the mean; SI: selectivity index*.

Chalcones are known for their simple chemistry, ease of synthesis, and suitability for derivatization. These compounds attracted attention due to a wide range of biological activities such as antidiabetic [33], antihypertensive [34], antiviral [35], anti‐inflammatory [36], antihistaminic [37], antioxidant [38], antimalarial [39], and anticancer [40]. Chalcones display notable anticancer activity through both structural features and cellular mechanisms [41]. 2′,4′‐dihydroxy‐3′‐methoxy‐3,4‐methylenedioxy‐8‐hydroxymethylene dihydrochalcone isolated from *Sansevieria cylindrica* Bojer ex Hook (Dracaenaceae) showed a moderate cytotoxic effect against MCF‐7 cells (IC_50_ = 34 µg/mL) [42]. Erioschalcone A isolated from *Eriosema glomeratum* (Guill. & Perr.) Hook.f. (Fabaceae) and its semi‐synthetic derivative 4‐(3‐(4‐methoxyphenyl)propanoyl)‐2‐(3‐methylbut‐2‐en‐1‐yl)‐1,3‐phenylenediacetate exhibited strong antiproliferative effect in A549, MCF‐7 and HeLA cells [43]. Inhibition of different targets such as aromatase, CDC25B, topoisomerase‐II, ABCG2/P‐gp/BCRP, 5α‐reductase, HDAC/Situin‐1, proteasome, VEGF, VEGFR‐2 kinase, 17‐β‐hydroxysteroid dehydrogenase, MMP‐2/9, JAK/STAT signaling pathways, tubulin, cathepsin‐K, Wnt, NF‐κB, B‐Raf, and mTOR played a role in the anticancer activity mechanism. Replacing the aryl ring with electron‐withdrawing/donating groups and/or replacing the aryl with a heteroaryl ring was found to increase the activity in structure‐anticancer activity studies [40, 41, 42, 43].

Chalcones isolated from Apiaceae species, such as those found in Ferula and related genera, display remarkable anticancer properties through multiple mechanisms of action. Prenylated chalcones, including kayserin A and B from F. caspica roots, have been shown to induce apoptosis in COLO 205, K‐562, and MCF‐7 cells via caspase‐3/8/9 activation and suppression of Bcl‐xL expression [44]. Similarly, the prenylated chalcone xanthohumol exhibits strong cytotoxic activity across prostate, breast, pancreatic, and leukemia cell lines; it promotes mitochondrial depolarization, upregulates ROS, triggers caspase cleavage, inhibits survival signaling (Akt, NF‐κB, mTOR), and disrupts VEGF‐driven angiogenesis in vitro and in tumor xenografts [45]. These bioactivities align with broader findings on structure‐activity relationship (SAR) in the Apiaceae family. Diprenylation often enhances lipophilicity and membrane affinity, which strengthens interaction with mitochondrial or signaling targets, while combinations of hydroxyl and methoxy substitutions modulate redox balance and pathway inhibition [46]. Collectively, chalcones from Apiaceae exert a polypharmacological approach, including apoptosis induction, ROS generation, survival‐pathway blockade, and anti‐angiogenic effects, that underscores their potential as promising leads in anticancer drug development.

The newly isolated chalcone‐type compound, 2',6'‐dihydroxy‐4'‐methoxy‐3''‐(γ,γ‐dimethylallyl)‐4''‐hydroxy β‐hydroxydihydrochalcone (compound **3**), incorporates several structural elements that are closely associated with anticancer activity according to SAR studies. Hydroxyl groups at the 2′ and 6′ positions enhance reactive oxygen species (ROS) formation and support mitochondrial apoptosis, as seen in other chalcone analogues [47]. The 4′‐methoxy group is known to improve membrane permeability and bioavailability without dramatically raising lipophilicity, consistent with findings in 2,4,5‐trimethoxy chalcone studies, where para electron‐donating substitutions increased cytotoxicity in MCF‐7, SW‐982, and HeLa cells [48]. Incorporation of a prenyl (γ,γ‐dimethylallyl) group at 3″ likely boosts lipophilicity and membrane localization, which has been shown to enhance selectivity and cell uptake . Yet, maintaining optimal lipophilicity is critical since SAR assessments revealed that excessive lipophilicity can impair cellular permeability in K562 and SH‐SY5Y models [49]. Although the β‐hydroxydihydrochalcone backbone lacks the usual α,β‐unsaturated carbonyl, the saturated β‐hydroxy form may confer increased metabolic stability while still engaging alternative targets. Methoxy and hydroxyl substitutions are also known to disrupt survival signaling via NF‐κB or PI3K/Akt pathways and to inhibit drug‐efflux transporters like ABCG2 with the 6′‐OH + 2′,4′‐OMe pattern identified as especially effective [47]. Collectively, these structural features suggest that compound **3** could act through mitochondrial apoptosis activation, redox modulation, signal‐pathway interference, and possible reversal of multidrug resistance, although confirmatory biochemical assays will be essential.

### 2.3 In Silico Analysis

Considering cytotoxic activity, according to the CLC‐Pred software, umbelliferone 6‐carboxylic acid (**1**) would not show cytotoxicity on any cell line. However, umbelliferone (**2**) may have cytotoxic activity against oligodendroglioma (Pa = 0.618; Pi = 0.021) and breast carcinoma (Pa = 0.504; Pi = 0.051). Nodakenetin (**4**) was estimated to be effective against promyeloblast leukemia (Pa = 0.524; Pi = 0.024) and small cell lung carcinoma (Pa = 0.508; Pi = 0.013). It was determined that compound **3** may show activity against small‐cell lung carcinoma (Pa = 0.547; Pi = 0.007), consistent with the results of our experimental study. For the new compound whose cytotoxic activity we identified in lung cancer cells, the first 10 genes listed for mRNA‐based up‐ and down‐regulation were determined (Table 5).

**TABLE 5:** Prediction results of gene expression alterations induced by the new compound **3**.

Up regulation			Down regulation		
Pa‐Pi	Genes	^*^IAP, LOO CV	Pa‐ Pi	Genes	^*^IAP, LOO CV
0.943‐0.007	PARP1	0.861	0.958‐0.003	HK2	0.816
0.925‐0.006	BIRC2	0.824	0.947‐0.004	PARP1	0.807
0.922‐0.004	SNORA74A	0.902	0.942‐0.004	ADD1	0.793
0.920‐0.004	ZBTB10	0.799	0.932‐0.004	IDNK	0.764
0.915‐0.004	SOD2	0.904	0.926‐0.003	LRG1	0.865
0.913‐0.005	HTRA2	0.762	0.923‐0.004	COX10	0.762
0.908‐0.002	EBLN2	0.910	0.916‐0.004	CHML	0.808
0.903‐0.004	AP5Z1	0.859	0.913‐0.003	SULT1A1	0.786
0.900‐0.009	MSANTD3	0.762	0.912‐0.005	PWP1	0.864
0.899‐0.004	FZD2	0.774	0.907‐0.004	ACE2	0.866

* IAP: invariant accuracy of prediction; LOO CV: leave‐one‐out cross‐validation.

Recently, the HK2 (hexokinase) gene has been shown to be a novel therapeutic target in lung cancer [50], and compound **3** was predicted as an inhibitor of HK2 in the current study. Investigating the interaction with this gene in future studies involving compound **3** would be beneficial. For the isolated compounds, the top 10 highest prediction scores among the different activities are given in Table 6.

**TABLE 6:** In silico biological activity results of isolated compounds.

Compound	Biological activities	Probability (P_active_‐P_inactive_)
**1**	4‐Nitrophenol 2‐monooxygenase inhibitor	0.936‐0.001
	CYP2C12 substrate	0.938‐0.005
	Chlordecone reductase inhibitor	0.926‐0.004
	Antiuremic	0.917‐0.002
	CYP2A11 substrate	0.912‐0.002
	Antiseptic	0.903‐0.003
	Glucan endo‐1,6‐beta‐glucosidase inhibitor	0.900‐0.003
	Aryl‐alcohol dehydrogenase (NADP+) inhibitor	0.896‐0.002
	UGT1A6 substrate	0.893‐0.003
	Cholestantriol 26‐monooxygenase inhibitor	0.885‐0.003
**2**	CYP2C12 substrate	0.976‐0.002
	CYP2A11 substrate	0.941‐0.001
	Chlordecone reductase inhibitor	0.936‐0.003
	Membrane integrity agonist	0.937‐0.004
	Aspulvinone dimethylallyl transferase	0.933‐0.004
	4‐Nitrophenol 2‐monooxygenase inhibitor	0.928‐0.002
	CYP2B5 substrate	0.915‐0.002
	CYP2A4 substrate	0.914‐0.001
	Cardiovascular analeptic	0.908‐0.003
	Aryl‐alcohol dehydrogenase (NADP+) inhibitor	0.906‐0.002
**3**	CDP‐glycerol glycerophosphotransferase inhibitor	0.881‐0.013
	Membrane integrity agonist	0.866‐0.020
	Ubiquinol‐cytochrome‐c reductase inhibitor	0.836‐0.021
	CYP2D6 substrate	0.817‐0.005
	UDP‐glucuronosyltransferase substrate	0.804‐0.006
	UDP‐glucuronosyltransferase substrate	0.804‐0.006
	Aspulvinone dimethylallyltransferase inhibitor	0.785‐0.038
	Reductive	0.740‐0.005
	Lactase inhibitor	0.733‐0.005
	Choleretic	0.720‐0.004
**4**	CYP2C12 substrate	0.912‐0.009
	Antiischemic, cerebral	0.871‐0.007
	CYP2A11 substrate	0.855‐0.003
	Spasmolytic	0.773‐0.005
	Anti‐inflammatory	0.759‐0.009
	Spasmolytic, urinary	0.755‐0.006
	4‐Nitrophenol 2‐monooxygenase inhibitor	0.751‐0.005
	CYP2A4 substrate	0.722‐0.005
	CYP2F1 substrate	0.703‐0.005
	CYP2A6 substrate	0.694‐0.010

On the other hand, Table 7 displayed the compounds with the five highest probability scores for adverse and toxic effects. In further studies, these compounds can be investigated in terms of biological activities that are determined to have a biological effect based on the results of in silico analysis.

**TABLE 7:** Possible adverse and toxic effects of isolated compounds.

Compound	Possible adverse and toxic effects	Probability (P_active_‐P_inactive_)
**1**	Hematemesis	0.891‐0.004
	Allergic contact dermatitis	0.872‐0.004
	Ulcer, aphthous	0.854‐0.008
	Toxic, vascular	0.840‐0.010
	Photoallergy dermatitis	0.814‐0.003
**2**	Hematemesis	0.880‐0.005
	Shivering	0.852‐0.018
	Toxic, vascular	0.835‐0.011
	Allergic contact dermatitis	0.784‐0.008
	Gastrointestinal hemorrhage	0.780‐0.009
**3**	Sensitization	0.763‐0.005
	Hepatotoxic	0.763‐0.025
	Panic	0.718‐0.016
	Edema	0.712‐0.019
	Dyspnea	0.711‐0.020
**4**	Sedative	0.566‐0.011
	Hepatitis	0.586‐0.061
	Hematemesis	0.535‐0.061
	Inflammation	0.518‐0.073
	Endocrine disruptor	0.476‐0.054

## 3 Conclusions

In the present study, four secondary metabolites, including a previously undescribed *β*‐hydroxydihydrochalcone (**3**), were isolated from the whole plant parts of *O. hispidus* via cytotoxic activity‐guided isolation. At the tested concentrations, umbelliferone (**2**), umbelliferone 6‐carboxylic acid (**1**), and nodakenetin (**4**) did not show antiproliferative activity on the A549, while compound **3** exerted significant activity on the lung cancer cells. Moreover, compound **3** had lower cytotoxicity against normal cells than doxorubicin. Computer‐aided cytotoxicity predictions were also found to be consistent with experimental analysis. Additionally, the prediction analysis indicates that compound **3** affects a gene identified as a new therapeutic target in lung cancer is a positive result for this compound. The relatively high selectivity of compound **3** indicates that this compound deserves further anticancer research. To better understand the therapeutic potential of compound **3**, additional studies are needed to clarify its molecular targets and elucidate its mechanism of action for evaluating therapeutic potential and safety.

## 4 Experimental

### 4.1 General Experimental Procedures

Column chromatography (CC) was carried out on silica gel 60 (0.063‐0.200 mm; Merck) and Sephadex LH‐20 (25‐100 µm; General Electric Healthcare). For the flash chromatography, Buchi pump modules (C‐601), control unit (C‐620), and fraction collector (C‐660) were utilized. Analytical and preparative thin layer chromatography (TLC) analyses were carried out on Aluminum TLC plate, silica gel coated with flourescent indicator F254 (Merck); the examination was established by spraying with 1% vanillin/sulphuric acid solution followed by heating at 105°C for 2–3 min, detected with an ultraviolet (UV) at 254 and 365 nm. ^1^H (500 MHz), ^13^C (100 MHz), COSY, HSQC, and HMBC NMR spectra were recorded on a Bruker AM in dimethyl sulfoxide (DMSO)‐d_6_ or CD_3_OD. Using the peaks of the remaining solvents (DMSO‐d*_6_*: ^1^H, 2.50 ppm; CD_3_OD: ^1^H, 3.31 ppm), the chemical shifts are given as *δ* values in parts per million (ppm). The coupling constants (*J*) were reported as Hz. The low‐resolution mass spectrometry (LRMS) and high‐resolution MS (HRMS) data were carried out by using an Agilent G6530B TOF/Q‐TOF mass spectrometer (Agilent Technologies, Santa Clara, CA, USA; negative mode; in *m/z*) and a Thermo Orbitrap Q‐Exactive mass spectrometer (Thermo Fisher Scientific Inc., Waltham, MA, USA), respectively. ECD and UV spectra of the new compound were recorded on a Jasco J‐815 CD spectrophotometer over the range of 200‐400 nm and a Shimadzu UV‐1800 UV‐Vis spectrophotometer, respectively. Chloroform was used as a blank both for ECD and UV spectra. Infrared (IR) was measured on a Perkin Elmer Spectrum 100 FTIR Spectrometer. Optical rotation was measured on KRÜSS Optronic (Germany).

### 4.2 Plant Material

*O. hispidus* (Friv.) Griseb. was collected from Seydişehir, Konya, in July 2020. The plant was identified by Prof. Dr. Osman Tugay from Selcuk University, Faculty of Pharmacy, Department of Pharmaceutical Botany. A voucher specimen (KNYA‐30.041) was deposited at the Herbarium of the Faculty of Science, Selcuk University, Konya, Türkiye.

### 4.3 Extraction and Isolation

The air‐dried whole plant parts of *O. hispidus* (1300 g) were powdered and macerated with methanol (5 × 10 L, 24 h). Following filtration, combined extracts were concentrated under reduced pressure. The crude MeOH extract (202.18 g) was suspended with 400 mL distilled water and fractionated with *n*‐hexane (10 × 600 mL), dichloromethane (8 × 600 mL), ethyl acetate (8 × 600 mL), and *n*‐butanol saturated with H_2_O (8 × 600 mL) in a separatory funnel by liquid‐liquid extraction. The crude MeOH extract and sub‐extracts were evaluated for their cytotoxicity by MTT assay. The active sub‐extracts, *n*‐hexane (OHNH‐***O*** *popanax* ***h*** *ispidus* **N**‐**H**exane) and dichloromethane (OHDCM‐***O*** *popanax* ***h*** *ispidus* **D**i**C**hloro**M**ethane) were purified by successive chromatographic methods.

OHNH (31.27 g) was fractionated over a Sephadex LH‐20 CC (5 × 70 cm) eluted with MeOH to yield four main fractions (OHNH Fr1‐Fr4). The activities of these main fractions were evaluated on A549 and Beas 2B cell lines. The most active fraction, OHNH Fr2 (12.5 g), was separated by SP‐LH 20 CC employing MeOH to give six subfractions: Fr 2a‐2f. Fr 2f (25.7 mg) submitted to SP LH‐20 CC eluted with MeOH and purified via preparative TLC using CHCl_3_:MeOH:H_2_O (70:30:3) as mobile phase to yield compound **1** (5.5 mg). The subfraction Fr 2d (340 mg) was applied to the SP‐LH 20 column by eluting with *s*‐Hex:EtOAc:MeOH (7:4:1) to afford Fr 2_d‐1_‐ 2_d‐5_. Fr 2_d‐3_ (37 mg) was further purified by preparative TLC with *s*‐Hex:EtOAc:MeOH (7:4:1) to give compound **2** (1.8 mg).

OHDCM (26.32 g) was applied over a Sephadex LH‐20 column eluted with MeOH to yield two main fractions: OHDCM Fr1 and OHDCM Fr2. The most active fraction, OHDCM Fr2 (3.5 g), was separated by Sigel column chromatography eluting with a stepwise *n*‐Hex:EtOAc:MeOH gradient to obtain Fr2a‐2o subfractions. Fr 2b (940 mg) was subjected to Sephadex LH‐20 and eluted with MeOH to obtain Fr 2_b‐1_‐2_b‐4_. Fr 2_b‐3_ was purified by preparative TLC using *n*‐Hex:EtOAc (70:40) to afford compound **3** (10 mg). The subfraction Fr 2i (90 mg) was separated by LiChroprep C18‐MPLC using a stepwise H_2_O:MeOH gradient (65:35→25:75) to yield 2_i‐1_‐2_i‐6_. Compound **4** (6.6 mg) was isolated from 2_i‐3_ using preparative TLC eluting CHCl_3_:MeOH:H_2_O (90:10:1).

### 4.4 Structure Elucidation

As a result of sequential chromatographic methods, two simple coumarins (**1**, **2**), a new *β*‐hydroxydihydrochalcone (**3**), and a furanocoumarin (**4**) were isolated from *O. hispidus*. The structures of the isolated compounds were determined through spectroscopic analysis, including MS, 1D‐/2D‐NMR. The comparison of spectroscopic data with literature led to the characterization of the structures as umbelliferone 6‐carboxylic acid (**1**) [51], umbelliferone (**2**) [51], and nodakenetin (**4**) [52].

**Compound 3**: Grayish white powder; [α]^20^_D_ = ‐148 (*c* = 0.02, MeOH); UV (MeOH) λmax 287 nm; ^1^H NMR (500 MHz in CD_3_OD) and ^13^C NMR (CD_3_OD, 125 MHz); HRESIMS (*m/z*) 371.1457 [M‐H]^‐^ (calcd. for C_21_H_23_O_6_, 371.1489) (Figures S6–S14; Table 1).

### 4.5 Cell Lines and Cell Culture

Adenocarcinomic human alveolar basal epithelial cell line (A549), human cervical cancer cell line (HeLa), human breast cancer cell line (MCF‐7), human hepatocellular carcinoma cell line (HepG2), and human bronchial epithelial cell line (BEAS‐2B) were used for the cytotoxicity test panel of crude extract. *O. hispidus* sub‐extracts showed high sensitivity in lung cancer cells. Therefore, A549 was used in further activity‐guided fractionation and isolation studies. The cell lines were cultured in Dulbecco's Modified Eagle Medium (DMEM) supplemented with 10% fetal bovine serum (FBS), 1% L‐glutamine, and 1% penicillin‐streptomycin. Cells were maintained in an incubator at 37°C under 5% CO_2_.

### 4.6 MTT Assay

MTT assay was used to determine the cytotoxic activity of the crude extract, subextracts, main fractions, and isolated compounds. MTT assay, called the “gold standard” of cell viability tests, is based on measuring metabolic activity. [53, 54] The cells reaching sufficient confluence were counted and plated in 96‐well plates (6  ×  10^3^ cells/well). After 24 h, cells were treated with five concentrations of the extracts (1–100 µg/mL), main fractions (1–100 µg/mL), or pure compounds (0.5–50 µM) in DMSO. Doxorubicin (10/3/1/0.3 µM) was used as a positive control. The plates were incubated with test materials for 48 h. After treatment, the supernatant was removed, and fresh medium and MTT solution (1 g/L in PBS) were added to each well. The cells were incubated for three h at 37°C. The resulting formazan crystals were dissolved in DMSO, and finally, the absorbance was detected at 570 nm using a spectrophotometer (SpectraMax i3x; Molecular Devices, San Jose, CA, USA). All data were collected from three independent experiments, and at least *n* = 6 data points were obtained. IC_50_ values were calculated as µg/mL for extracts or fractions and as µM for pure compounds.

### 4.7 Selectivity Index

The selectivity index (SI), which indicates the selectivity between cancer and normal cells, was calculated. The compounds with an SI greater than 1 are more likely to inhibit cancer cells than non‐cancerous cells [55]. SI values were calculated by the formula of [(IC_50_ values of non‐cancerous cells)/(IC_50_ values of cancerous cells)].

### 4.8 In Silico Analysis

The isolated compounds were evaluated in terms of anticancer and different biological activities by *in silico* analysis. For this purpose, PASS (Prediction of Activity Spectra for Substances) and CLC‐Pred (Cell Line Cytotoxicity Predictor) programs were used [56]. PASS evaluates the overall biological activity potential of tested molecules. It predicts more than 4000 activities. In this software, Pa and Pi values represent high and low probabilities of being active or inactive, respectively. In silico prediction of the cytotoxicity was performed using CLC‐Pred (Cell Line Cytotoxicity Predictor [2.0]) [57]. Additionally, the DIGEP‐Pred 2.0 (prediction of drug‐induced changes of gene expression profile) program was utilized to understand the alterations in gene expression related to the identified cytotoxic compound.

### 4.9 ECD Computational Analysis

The initial conformational analysis of compound **3** was performed using the semi‐empirical PM3 method [58] with the aid of the SPARTAN’04 program package. The resulting minimum‐energy conformers were subsequently optimized using DFT at the B3LYP/6‐31G(d) level, as implemented in the Gaussian 09 software package [59].

The predominant conformers were then subjected to theoretical ECD calculations using TDDFT at the B3LYP/6‐311G(d,p) level in chloroform [60, 61], modeled using the conductor‐like polarizable continuum model (CPCM) [62]. The energies, oscillator strengths, and rotational strengths of each conformer were calculated with the Gaussian 09 software package. The theoretical ECD spectra of each conformer were simulated using Gaussian band‐shape functions.

### 4.10 Statistical Analysis

Data were analyzed using GraphPad Software Prism 8.0 (San Diego, CA, USA; demo version). The significance of differences in means of the cell viability % of each extract, fraction, and isolated compounds on cells was determined by one‐way analysis of variance (ANOVA), followed by Tukey's multiple comparison tests. Nonlinear regression analysis (dose‐response) was used to determine the IC_50_ values. *p* < 0.05 was considered statistically significant.

## Author Contributions

**Kevser Taban**: writing original draft, methodology, investigation, formal analysis, and data curation. **İpek Süntar**: supervision, methodology, investigation, formal analysis, data curation, conceptualization, writing – review and editing. **Perihan Gürbüz**: methodology, investigation, data curation, writing – review and editing. **Esra Emerce**: validation, software, and methodology. **Osman Tugay**: investigation and resources. **Şengül Dilem Doğan**: methodology, investigation, and data curation. **Beyza Hamur**: investigation and formal analysis. **Ahmet Ceyhan Gören**: investigation and formal analysis.

## Conflicts of Interest

The authors declare no conflict of interest.

## Supporting information




**Supporting File 1**: cbdv70254‐sup‐0001‐SupMat.pdf.

## Data Availability

The data that support the findings of this study are available from the corresponding author upon reasonable request.
