# Supplementary material for: Cytotoxic Activity of Opopanax hispidus (Friv.) Griseb.: Characterization of a New Chalcone‐type Compound
Source: Chem Biodivers. 2025 Jul 22;22(11):e01077. doi: 10.1002/cbdv.202501077 (PMC12629158; doi:10.1002/cbdv.202501077)
Supplement: Supplementary file 1 — Supporting File 1: cbdv70254‐sup‐0001‐SupMat.pdf. [file CBDV-22-e01077-s001.pdf]

Supporting Information

	Table of Contents	Page
**FIGURE S1**	Cytotoxic effects of methanol extract of *O. hispidus* and doxorubicin on the tested cell lines	**2**
**FIGURE S2**	Cytotoxic effects of *O. hispidus* sub-extracts and doxorubicin on the tested cell lines	**3**
**FIGURE S3**	Cytotoxic effects of fractions obtained from n-hexane sub-extract of *O. hispidus* and doxorubicin on the tested cell lines	**4**
**FIGURE S4**	Cytotoxic effects of fractions obtained from dichloromethane subextract of *O. hispidus* and doxorubicin on the tested cell lines	**5**
**FIGURE S5**	Cytotoxic effects of compounds isolated from active fractions of *O. hispidus* and doxorubicin on tested cell lines	**6**
**FIGURE S6**	Chemical structure and key ^{1}H-^{1}H COSY ( ) ve HMBC (H C) correlations for compound **3**	**7**
**FIGURE S7**	HR-ESI-MS Spectrum of compound **3** (negative mode)	**7**
**FIGURE S8**	^{1}H NMR spectrum of compound **3** (in CD_3OD, ^{1}H:500 MHz).	**8**
**FIGURE S9**	^{13}C NMR spectrum of compound **3** (in CD_3OD, ^{13}C: 125 MHz).	**9**
**FIGURE S10**	HMQC spectrum of compound **3.**	**10**
**FIGURE S11**	COSY spectra of compound **3**	**11**
**FIGURE S12**	HMBC spectra of compound **3**	**12**
**FIGURE S13**	UV-Vis spectrum of compound 3	**13**
**FIGURE S14**	FTIR spectrum of compound 3	**13**

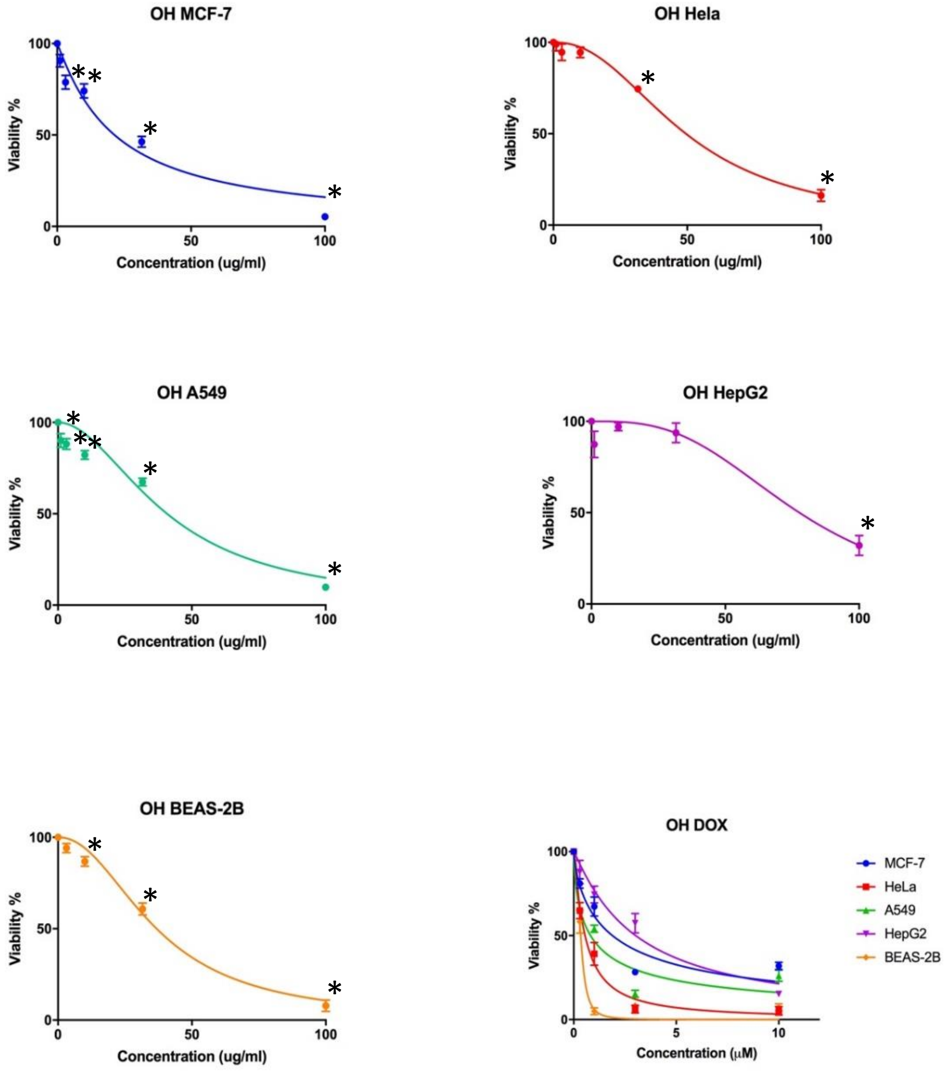

FIGURE S1 Cytotoxic effects of methanol extract of *O. hispidus* and doxorubicin on the tested cell lines (% mean cell viability ± SEM) OH: *O. hispidus*; Dox: Doxorubicin; SEM: Standard Error of Mean; *: Statistically significant compared to control (One way-ANOVA)

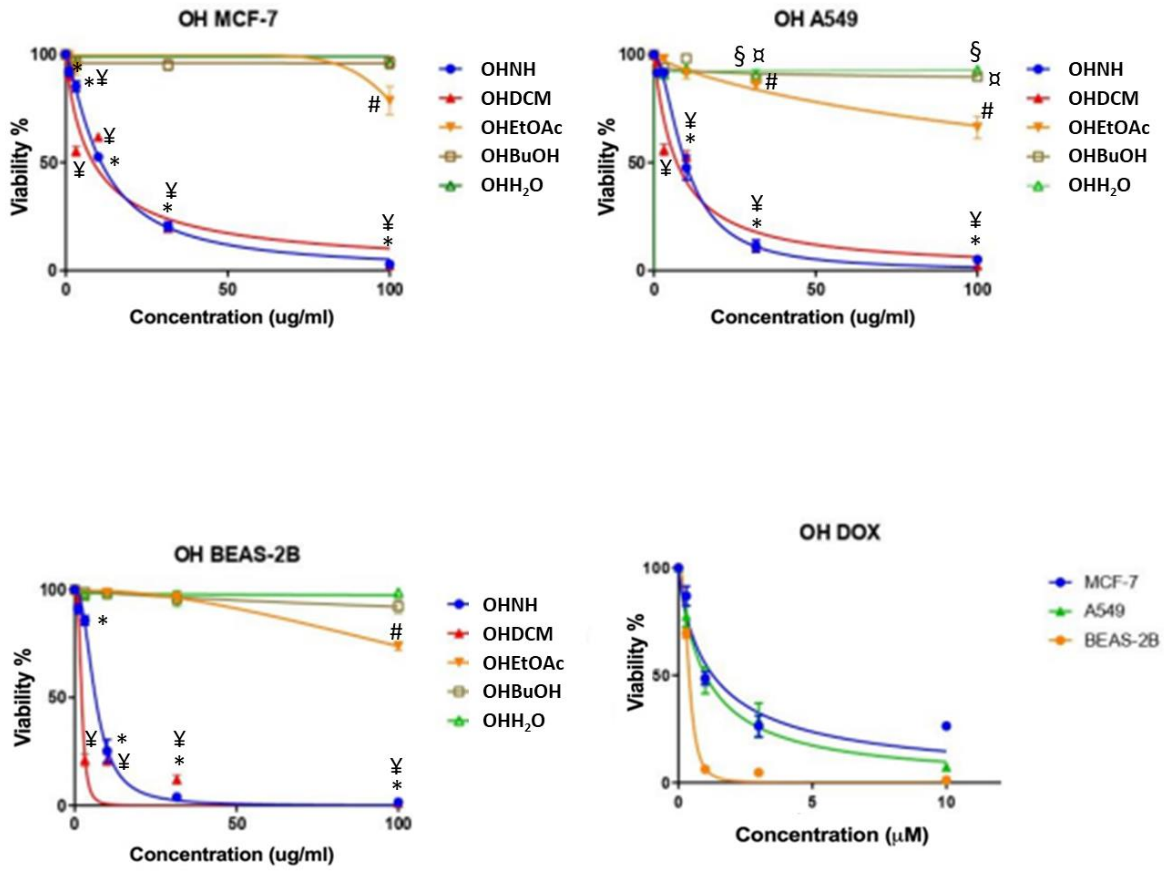

FIGURE S2 Cytotoxic effects of *O. hispidus* sub-extracts and doxorubicin on the tested cell lines (% mean cell viability ± SEM) OH: *O. hispidus*; Dox: Doxorubicin; OHNH: n-hexane; OHDCM: dichloromethane; OHEtOAc: ethyl acetate; OHBuOH: n-butanol; OHH_2O: water; SEM: Standard Error of Mean; *: Statistically significant compared to control for OHNH (One way-ANOVA); ¥: Statistically significant compared to control for OHDCM (One way-ANOVA); #: Statistically significant compared to control for OHEtOAc (One way-ANOVA); ¤: Statistically significant compared to control for OHBuOH; §: Statistically significant compared to control for OHH_2O.

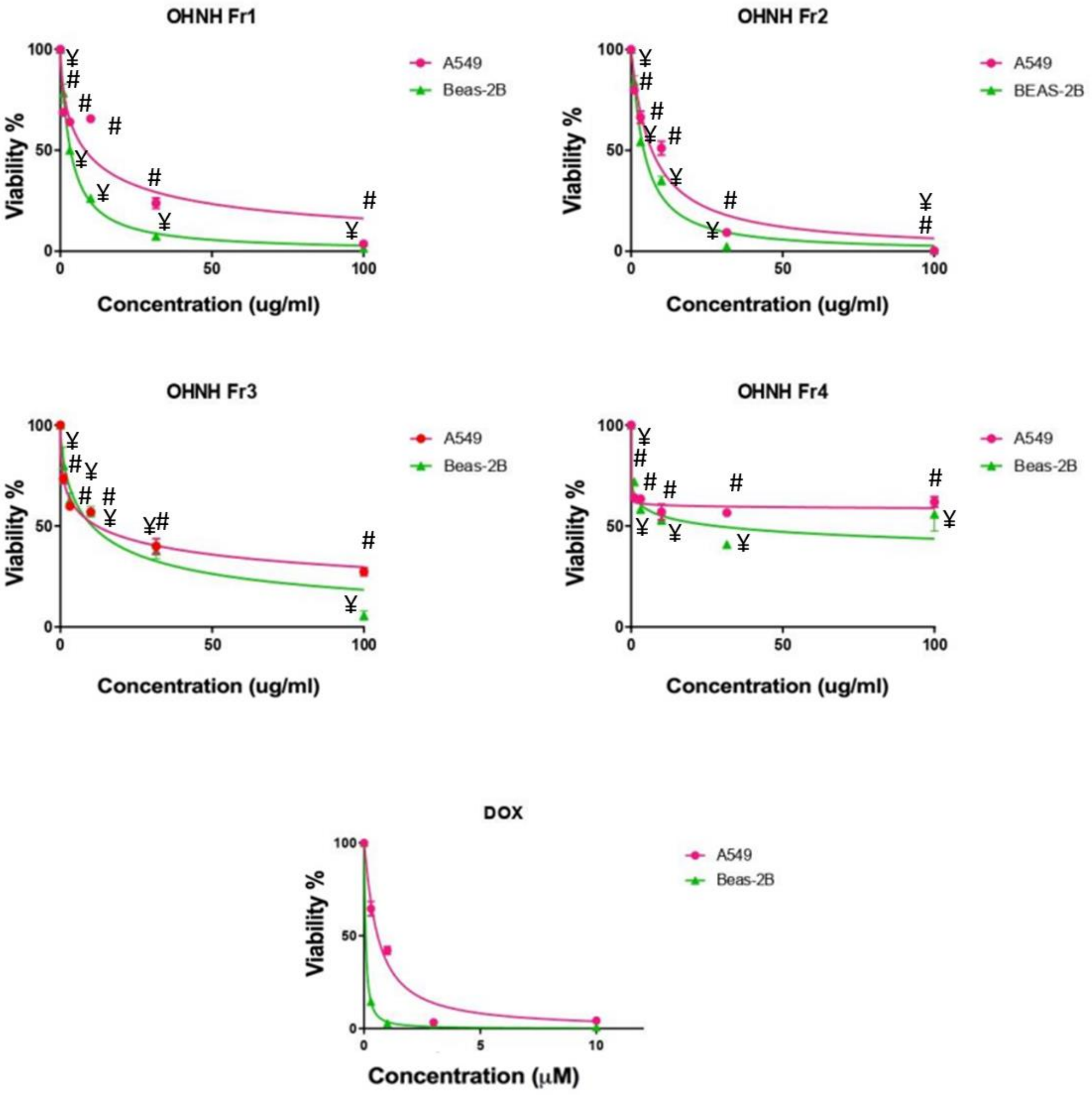

FIGURE S3 Cytotoxic effects of fractions obtained from n-hexane sub-extract of *O. hispidus* and doxorubicin on the tested cell lines (% mean cell viability ± SEM) OH: *O. hispidus*; Dox: Doxorubicin; *: Statistically significant compared to control for A549 (One way-ANOVA); ¥: Statistically significant compared to control for BEAS-2B (One way-ANOVA).

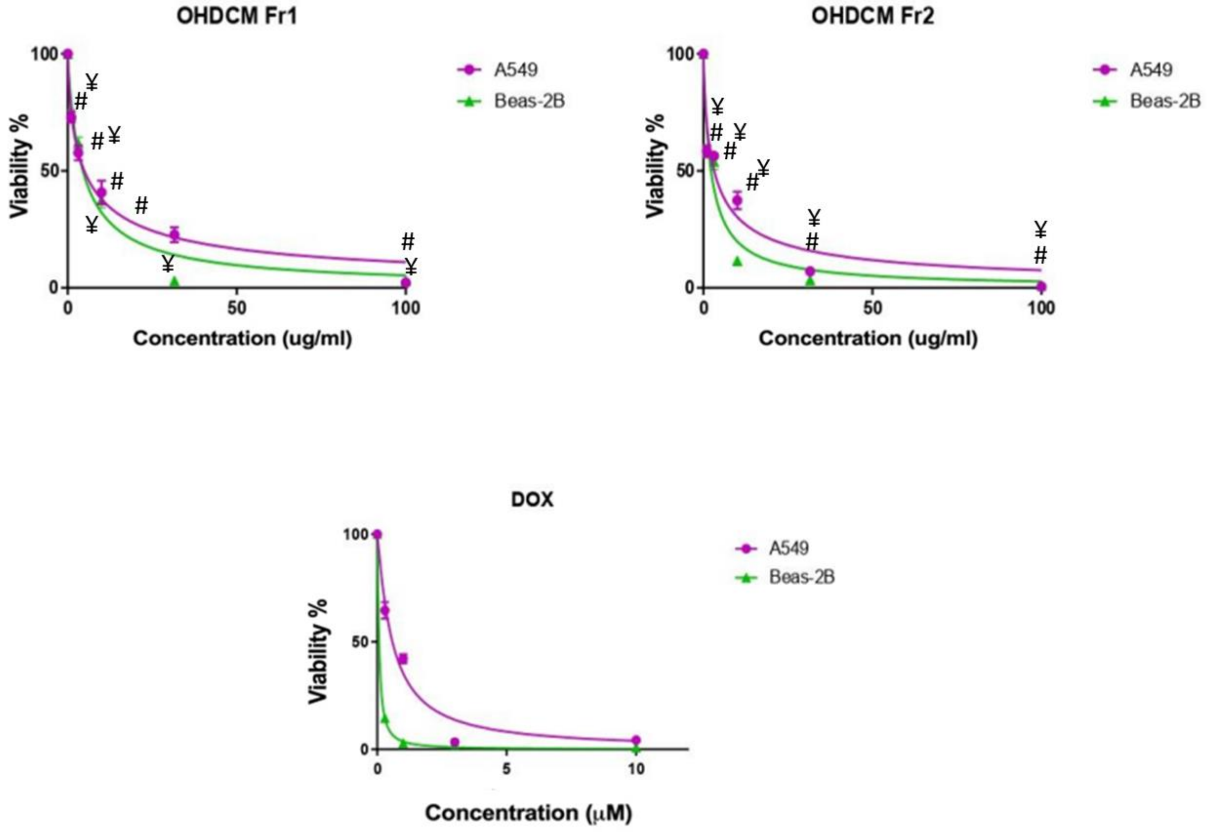

FIGURE S4 Cytotoxic effects of fractions obtained from dichloromethane subextract of *O. hispidus* and doxorubicin on the tested cell lines OH: *O. hispidus*; Dox: Doxorubicin; *: Statistically significant compared to control for A549 (One way-ANOVA); ¥: Statistically significant compared to control for BEAS-2B (One way-ANOVA).

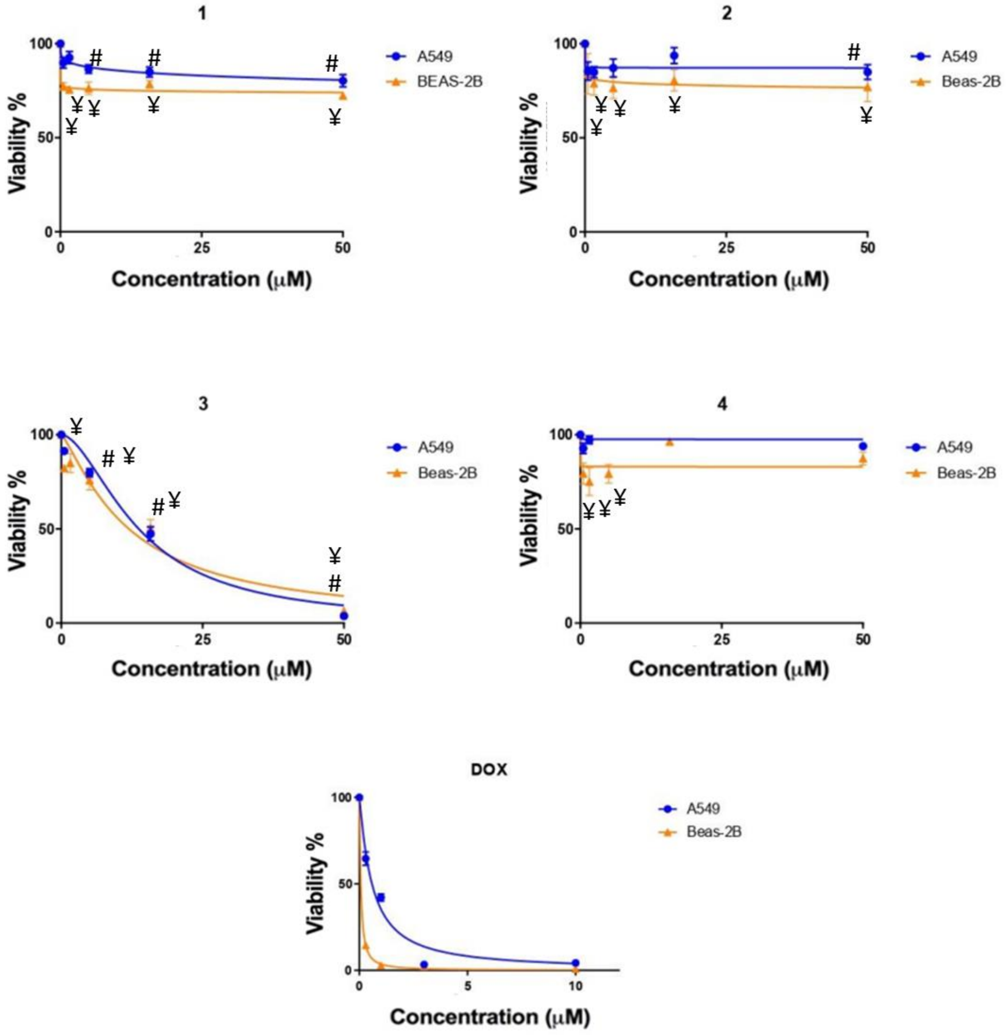

FIGURE S5 Cytotoxic effects of compounds isolated from active fractions of *O. hispidus* and doxorubicin on tested cell lines (% mean cell viability ± SEM) Dox: Doxorubicin; 1: Umbelliferone 6-carboxylic acid; 2: Umbelliferone; 3: 2',6'-dihydroxy-4'-methoxy-3''-(ϒ,ϒ-dimethylallyl)-4''-hydroxy β-hydroxydihydrochalcone ; 4: Nodakenetin.

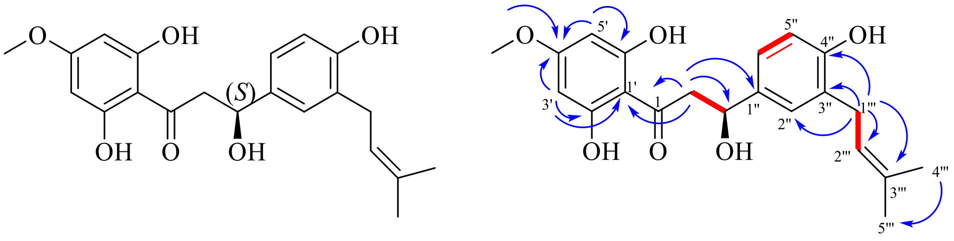

FIGURE S6 Chemical structure and key ^{1}H-^{1}H COSY (▬) ve HMBC (H ⌒ C) correlations for compound **3**

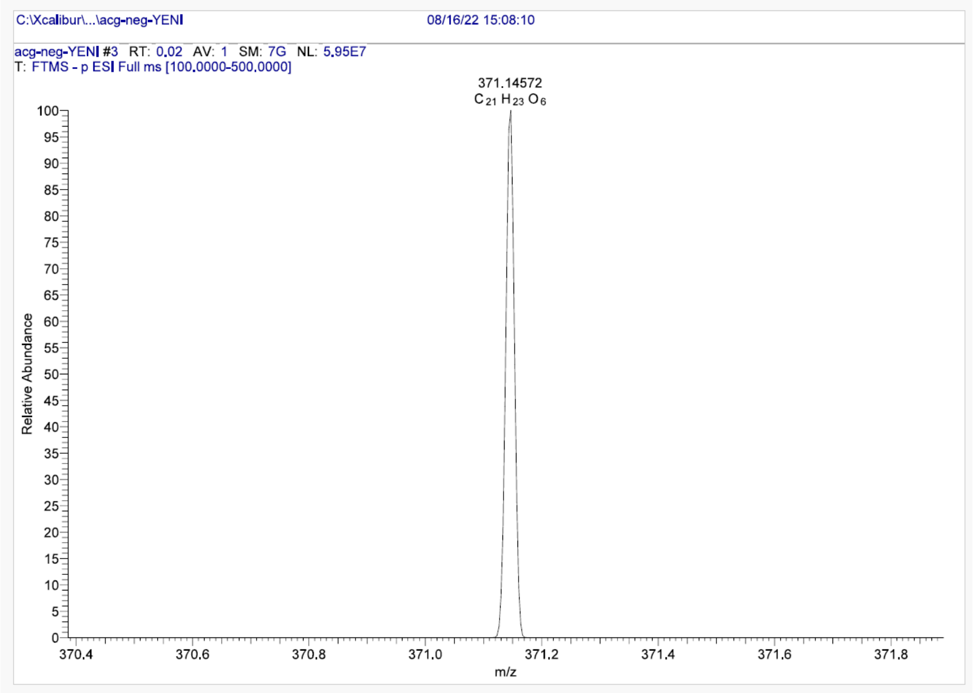

FIGURE S7 HR-ESI-MS Spectrum of compound **3** (negative mode)

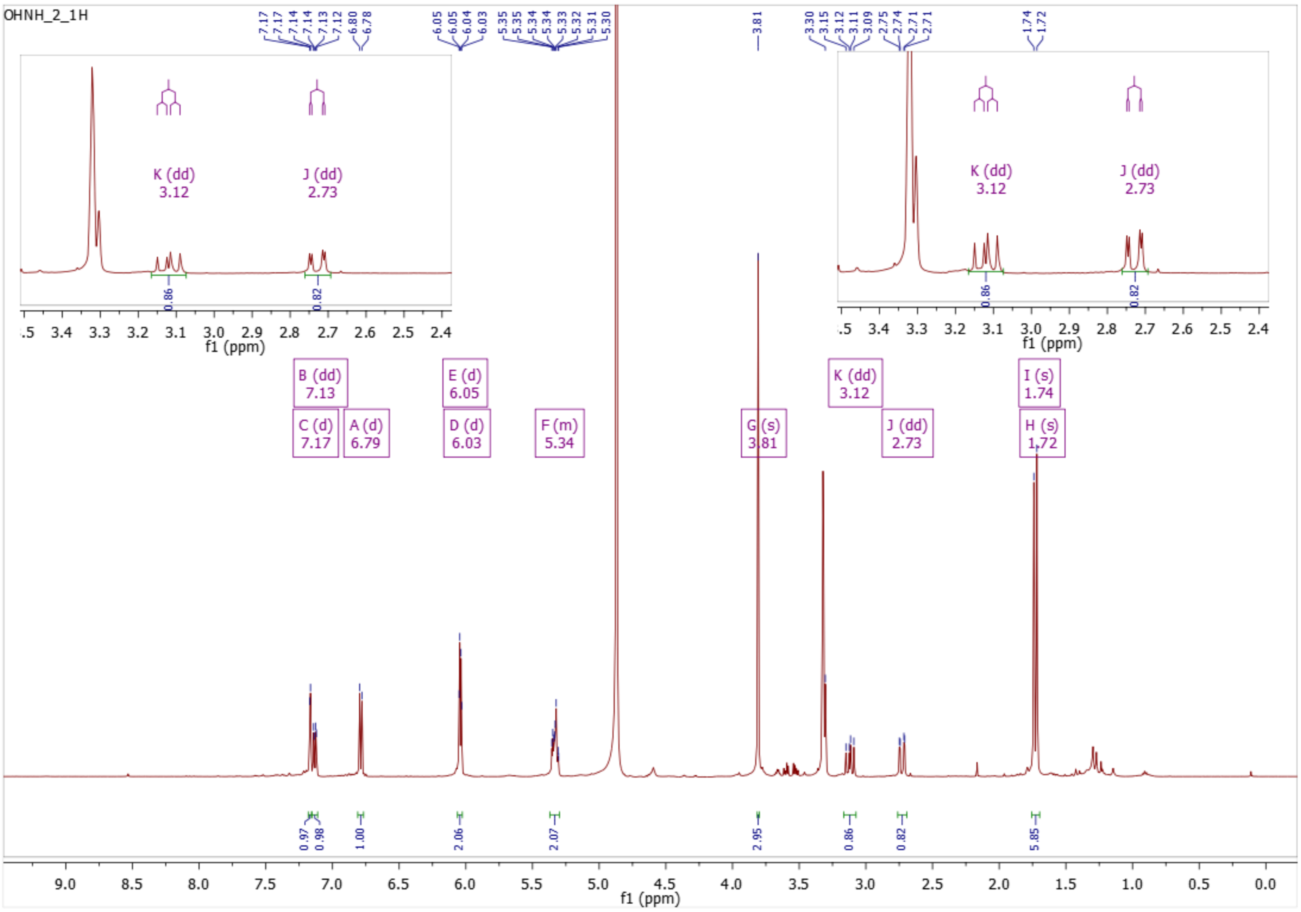

FIGURE S8 ^{1}H NMR spectrum of compound **3** (in CD_3OD, ^{1}H:500 MHz)

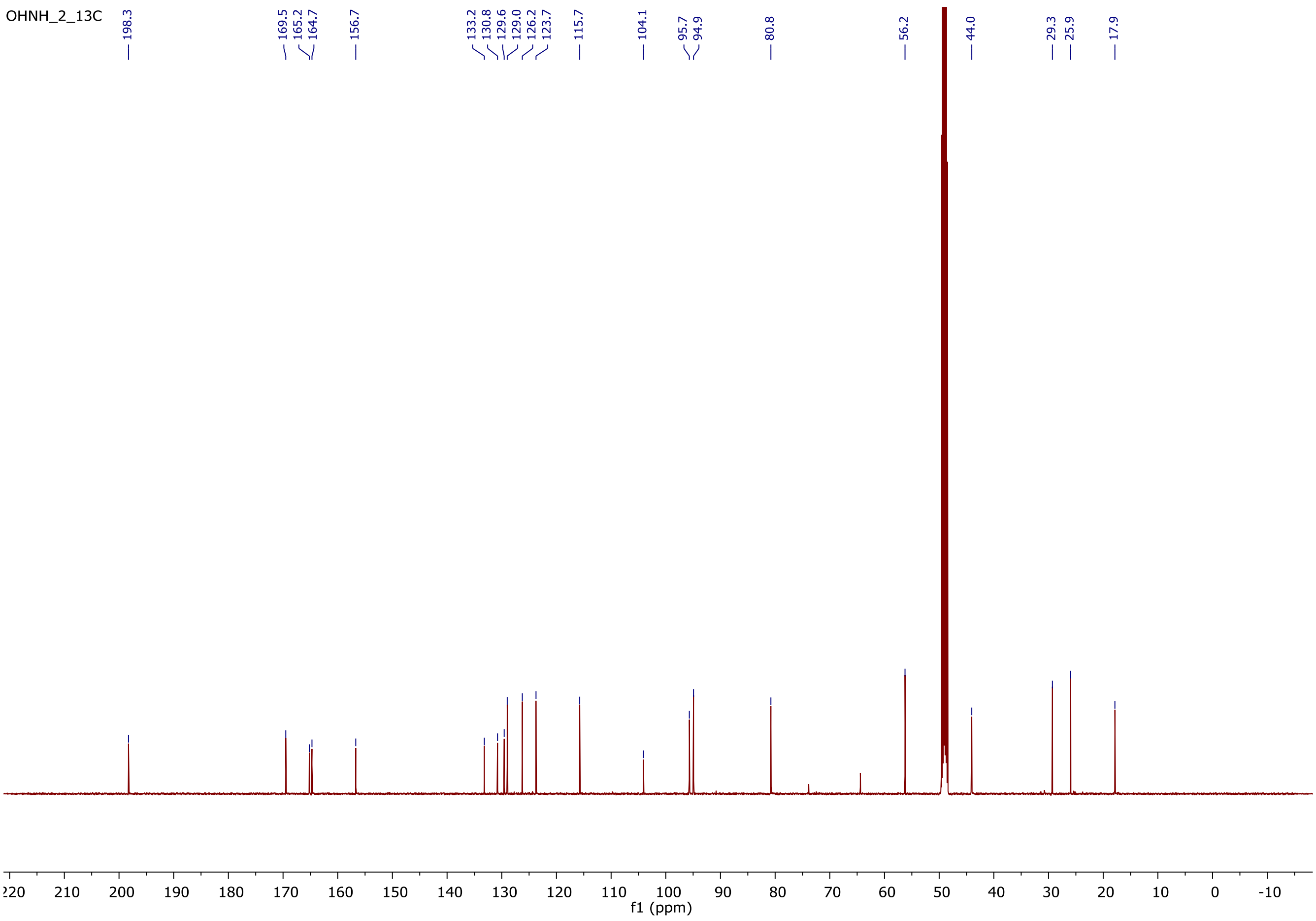

FIGURE S9 ^{13}C NMR spectrum of compound **3** (in CD_3OD, ^{13}C 125: MHz)

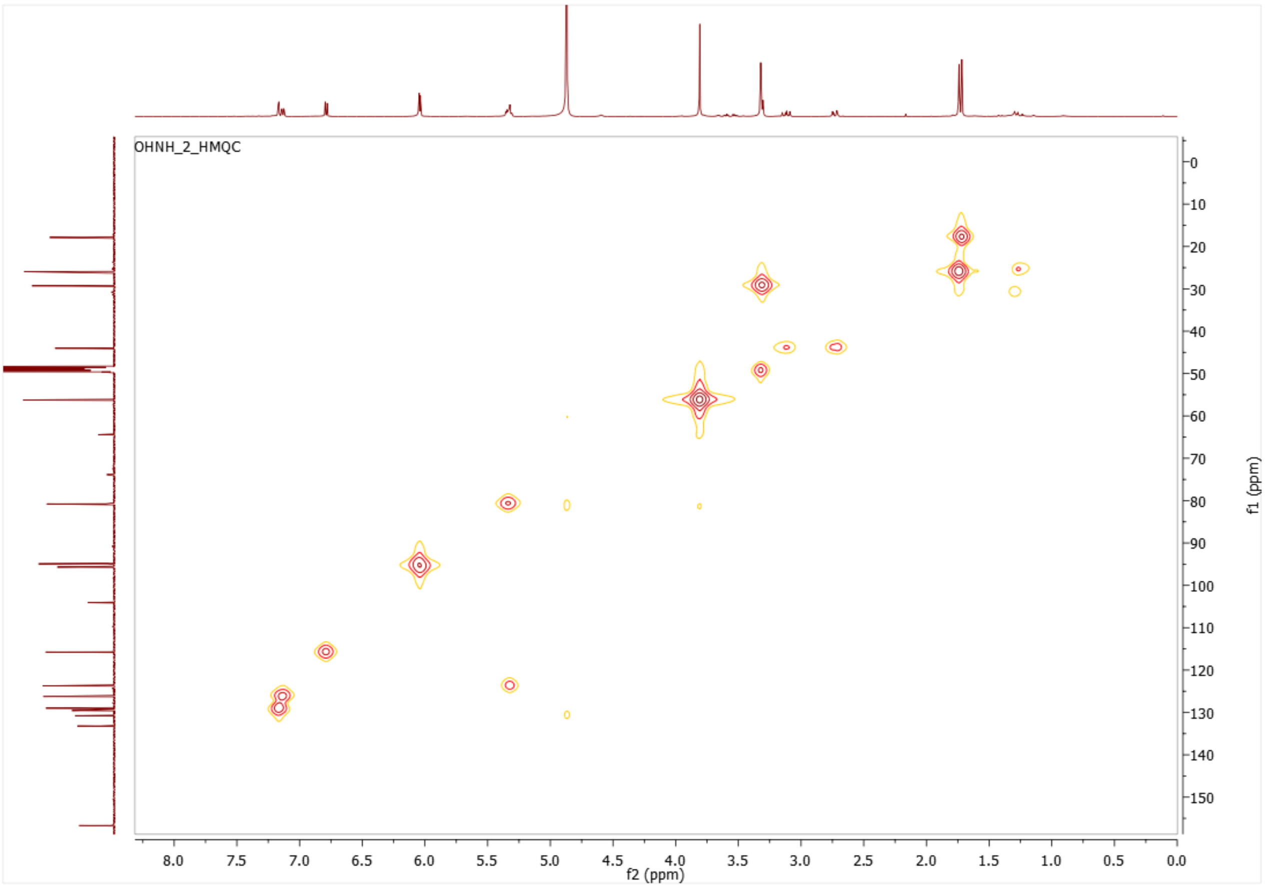

FIGURE S10 HMQC spectrum of compound **3**

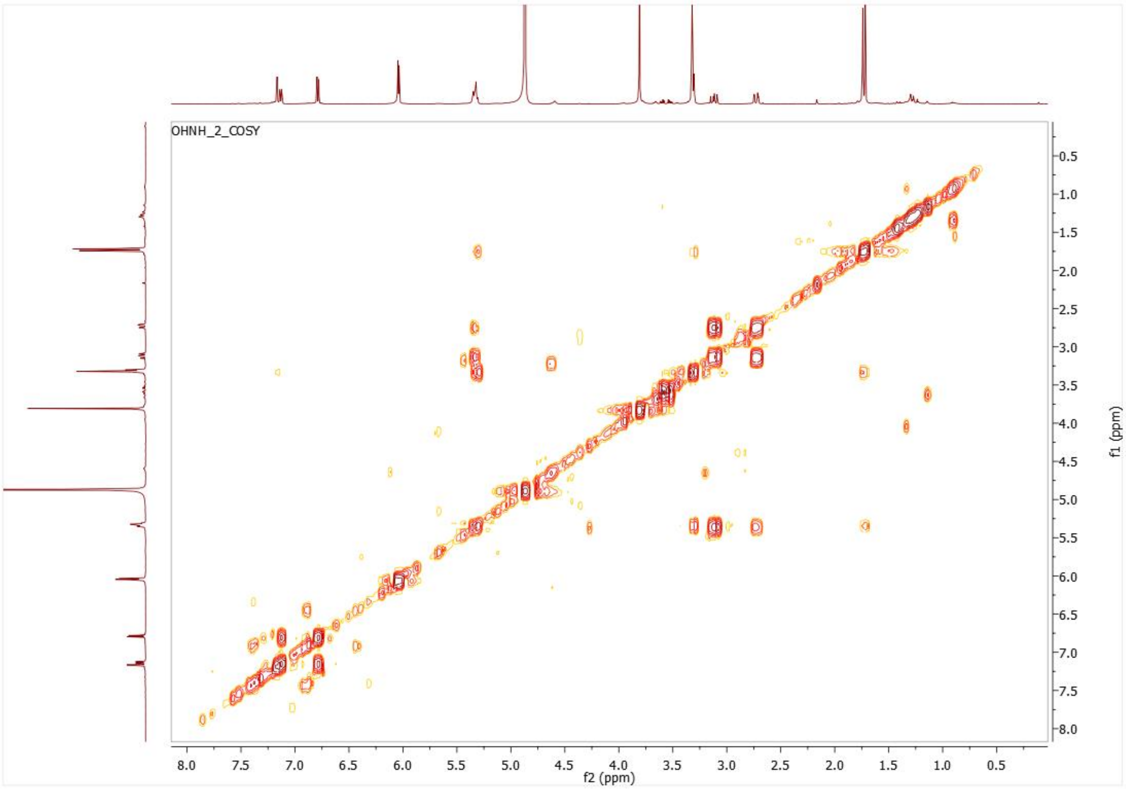

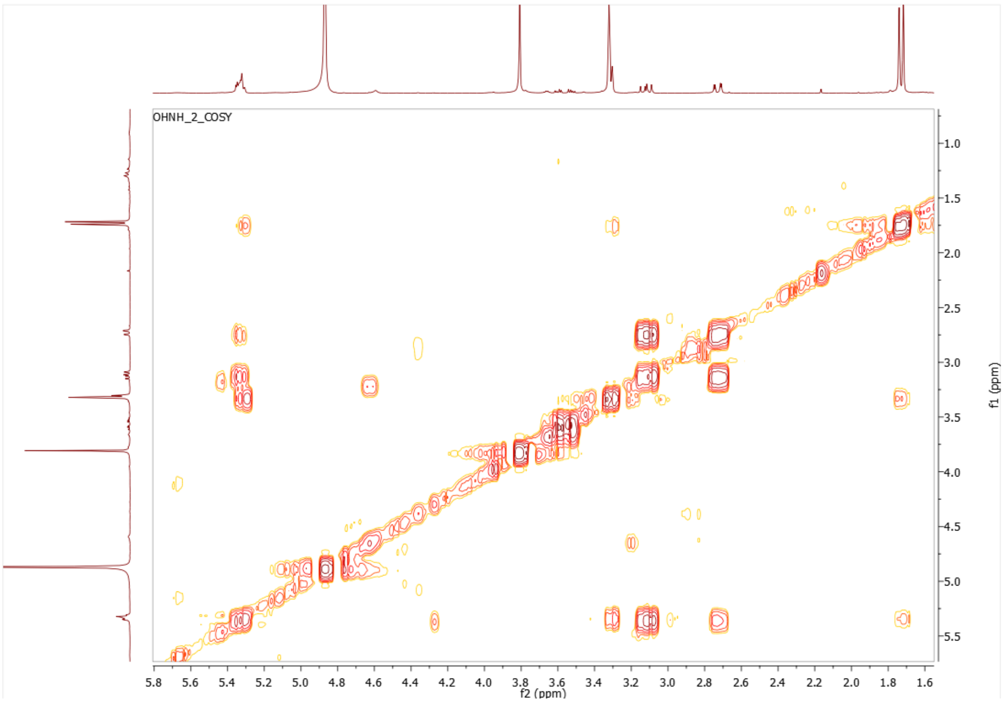

FIGURE S11 COSY spectra of compound **3**

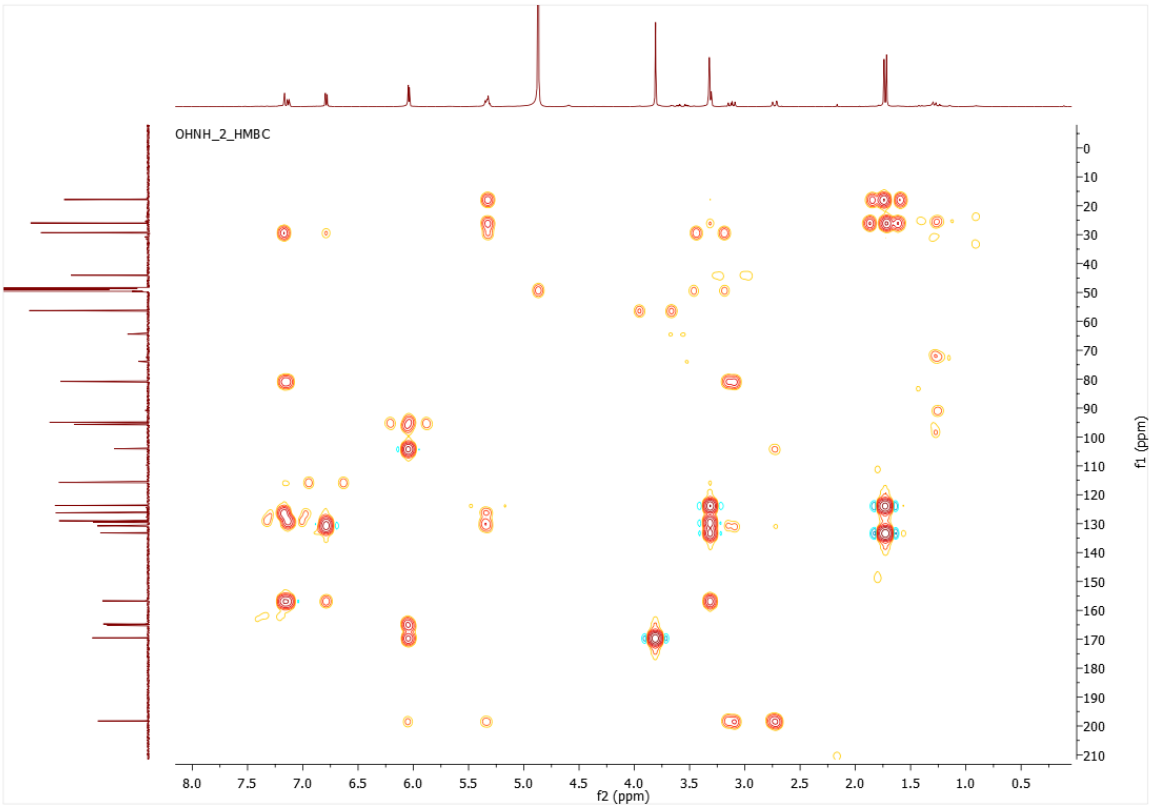

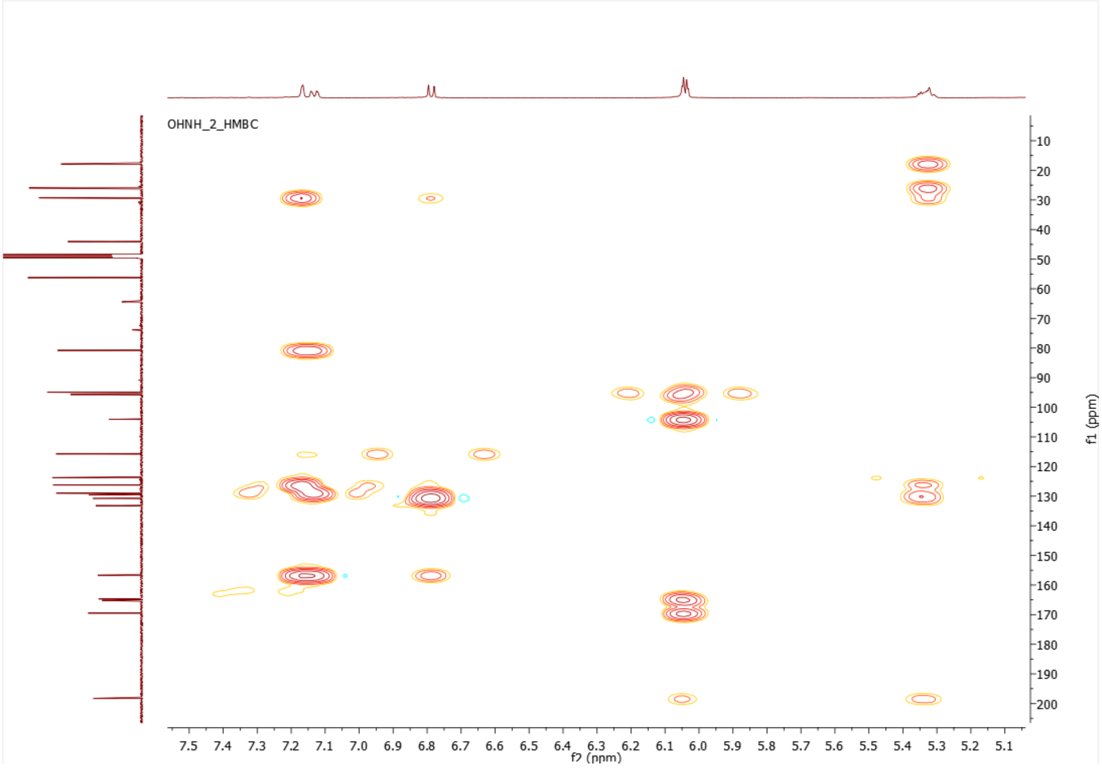

FIGURE S12 HMBC spectra of compound **3**

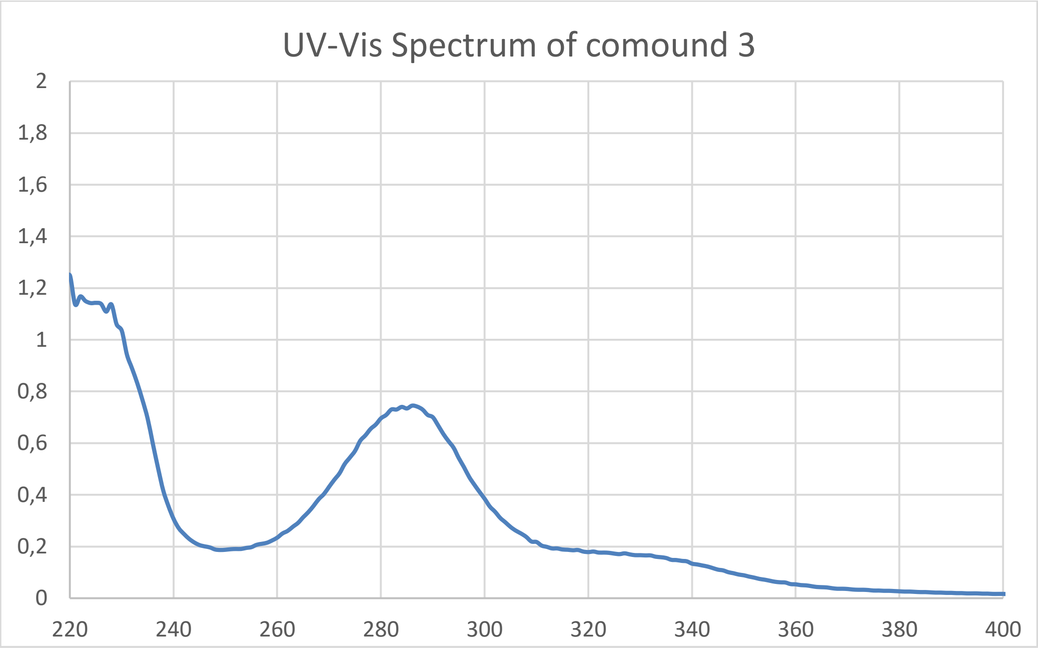

Figure S13 UV-Vis spectrum of Compound 3

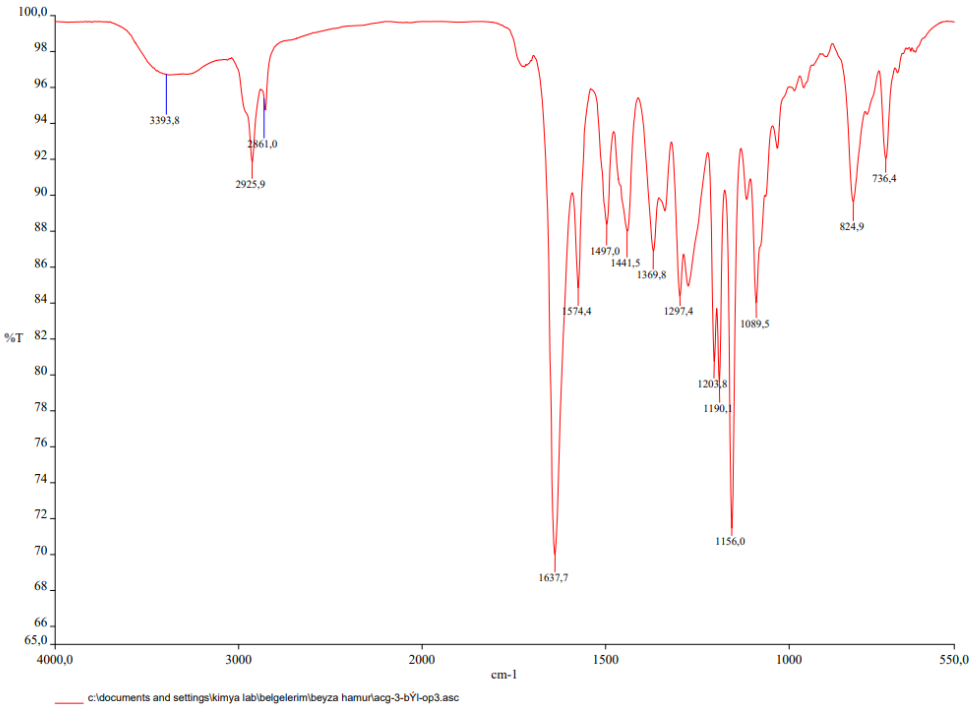

Figure S14 FTIR spectrum of Compound 3
